# Supplementary material for: The Relationship between Parenting Behaviors and Adolescent Well-Being Varies with the Consistency of Parent–Adolescent Cultural Orientation
Source: Behav Sci (Basel). 2024 Feb 28;14(3):193. doi: 10.3390/bs14030193 (PMC10967969; doi:10.3390/bs14030193)
Supplement: Supplementary file 1 [file behavsci-14-00193-s001.zip › behavsci-2841801-supplementary materials.pdf]

Sources of cultural orientation questionnaire

A. Individualism and Collectivism Domains Assessed in Individualism-Collectivism Scales[1]

Domain name	Description	Sample item
Individualism		
Independent	Freedom, self-sufficiency, and control over one's life	I tend to do my own thing, and others in my family do the same.
Goals	Striving for one's own goals, desires, and achievements	I take great pride in accomplishing what no one else can accomplish.
Compete	Personal competition and winning	It is important to me that I perform better than others on a task.
Unique	Focus on one's unique, idiosyncratic qualities	I am unique-different from others in many respects.
Private	Thoughts and actions private from others	I like my privacy.
Self-know	Knowing oneself; having a strong identity	I know my weaknesses and strengths.
Direct communicate	Clearly articulating one's wants and needs	I always state my opinions very clearly.
Collectivism		
Related	Considering close others an integral part of the self	To understand who I am, you must see me with members of my group.
Belong	Wanting to belong to and enjoy being part of groups	To me, pleasure is spending time with others.
Duty	The duties and sacrifices being a group member entails	I would help, within my means, if a relative were in financial difficulty.
Harmony	Concern for group harmony and that groups get along	I make an effort to avoid disagreements with my group members.
Advice	Turning to close others for decision help	Before making a decision, I always consult with others.
Context	Self-changes according to context or situation	How I behave depends on who I am With, where I am, or both.
Hierarchy	Focus on hierarchy and status issues	I have respect for the authority figures with whom I interact.
Group	A preference for group work	I would rather do a group paper or lab than do one alone.

B. INDCOL Scale [2]

SPOUSE

Sl. If a husband is a sports fan, a wife should also cultivate an interest in sports. If the husband is a stock broker, the wife should also be aware of the current market situation.

S2. A marriage becomes a model for us when the husband loves what the wife loves, and hates what the wife hates.

S3. Married people should have some time to be alone from each other everyday, undisturbed by

their spouse.
S4. If one is interested in a job about which the spouse is not very enthusiastic, one should apply for it anyway.
S.5. Even if my spouse were of a different religion, there would not be any interpersonal conflict between us.
S6. It is better for a husband and wife to have their own bank accounts rather than to have a joint account.
S7. The decision of where one is to work should be jointly made with one's spouse, if one is married.
S8. It is desirable that a husband and a wife have their own sets of friends, instead of having only a common set of friends.

PARENT
PI. My musical interests are extremely different from my parents.
P2. In these days parents are too stringent with their kids, stunting the development of initiative.
P3. When making important decisions, I seldom consider the positive and negative effects my decisions have on my father.
P4. Teenagers should listen to their parents' advice on dating.
P5. Even if the child won the Nobel prize, the parents should not feel honored in any way.
P6. It is reasonable for a son to continue his father's business.
P7. I would not share my ideas and newly acquired knowledge with my parents.
P8. I practice the religion of my parents.
P9. I would not let my needy mother use the money that I have saved by living a less than luxurious life.
P10. I would not let my parents use my car (if I have one), whether they are good drivers or not.
P11. Children should not feel honored even if the father were highly praised and given an award by a government official for his contribution and service to the community.
P12. Success and failure in my academic work and career are closely tied to the nurture provided by my parents.
P13. Young people should take into consideration their parents' advice when making education/career plans.
P14. The bigger a family, the more family problems there are.
P15. I have never told my parents the number of sons I want to have.
P16. The number of sons my parents would like me to have differs by [0 / 1 / 2 / 3 / 4 or more / I don't know] from the number I personally would like to have.

KIN
Kl. I would help, within my means, if a relative told me that he/she is in financial difficulty.
K2. If I met a person whose last name was the same as mine, I would start wondering whether we were, at least remotely, related by blood.
K3. Whether one spends an income extravagantly or stingily is of no concern to one's relatives (cousins, uncles).
K4. I would not let my cousin use my car (if I have one).
K5. When deciding what kind of work to do, I would definitely pay attention to the views of

relatives of my generation.
K6. When deciding what kind of education to have, I would pay absolutely no attention to my uncles' advice.
K7. Each family has its own problems unique to itself. It does not help to tell relatives about one's problems.
K8. I can count on my relatives for help if I find myself in any kind of trouble.

NEIGHBOR
N1. I have never chatted with my neighbors about the political future of this state.
N2. I am often influenced by the moods of my neighbors.
N3. My neighbors always tell me interesting stories that have happened around them.
N4. I am not interested in knowing what my neighbors are really like.
N5. One need not worry about what the neighbors say about whom one should marry.
N6. I enjoy meeting and talking to my neighbors every-day.
N7. In the past, my neighbors have never borrowed anything from me or my family.
N8. One needs to be cautious in talking with neighbors. Otherwise, others might think you are nosy.
N9. I don't really know how to befriend my neighbors.
Nl0. I feel uneasy when my neighbors do not greet me when we come across each other.

FRIEND
Fl. I would rather struggle through a personal problem by myself than discuss it with my friends.
F2. If possible, I would like co-owning a car with my close friends, so that it wouldn't be necessary for them to spend much money to buy their own cars.
F3. I like to live close to my good friends.
F4. My good friends and I agree on the best places to shop.
F5. I would pay absolutely no attention to my close friends' views when deciding what kind of work to do.
F6. To go on a trip with friends makes one less free and mobile. As a result, there is less fun.
F7. It is a personal matter whether I worship money or not. Therefore, it is not necessary for my friends to give any counsel.
F8. The motto "sharing in both blessing and calamity" is still applicable even if one's friend is clumsy, dumb, and causes a lot of trouble.
F9. There are approximately [0 / 1 / 2 / 3 / 4/ more than 4] of my friends who know how much my family as a whole earns each month.
F10. On the average, my friends' ideal number of children differs from my own ideal by [0 / 1 / 2 / 3 /4 or more / I don't know my friends' ideal].

CO-WORKER
Cl. It is inappropriate for a supervisor to ask subordinates about their personal life (such as where one plans to go for the next vacation).
C2. When I am among my colleagues/classmates, I do my own thing without minding about them.
C3. One needs to return a favor if a colleague lends a helping hand.
C4. I have never loaned my camera/coat to any colleagues/classmates.

C5. We ought to develop the character of independence among students. so that they do not rely upon other students' help in their schoolwork.
C6. A group of people at their workplace was discussing where to eat. A popular choice was a restaurant which had recently opened. However. someone in the group had discovered that the food there was unpalatable. Yet the group disregarded this person's objection, and insisted on trying it out. There were only two alternatives for the person who objected: either to go or not to go with the others. In this situation, not going with others is a better choice.
C7. There is everything to gain and nothing to lose for classmates to group themselves for study and discussion.
C8. Classmates' assistance is indispensable to getting a good grade at school.
C9. I would help if a colleague at work told me that he/she needed money to pay utility.
C10. In most cases, to cooperate with someone whose ability is lower than one's own is not as desirable as doing the thing alone.
C11. Do you agree with the proverb "Too many cooks spoil the broth"?

C. The "training" questionnaire[3]
Ideologies of child development and learning:
Children are by nature born good.
* Parents must begin training child as soon as ready.
Children can improve in almost anything if they work hard.
* Mothers must train child to work very hard and be disciplined.
* Mothers teach child by pointing out good behavior in others.
The best way child learns how to behave is to be around adults.
*When child continues to disobey you, he/she deserves a spanking.

Ideologies of the mother-child relationship:
*Mothers primarily express love by helping child succeed, esp. in school.
A mother's sole interest is in taking care of her child.
* Child should be in the constant care of their mothers or family.
Mothers should do everything for child's education and make many sacrifices.
* Child should be allowed to sleep in mother's bed.
* Child should be able to be with mother and taken on errands and gatherings.

D. Vertical and Horizontal Individualism and Collectivism Items[4]
Horizontal individualism(H-I)
I often do 'my own thing.'
One should live one's life independently of others.
I like my privacy.
I prefer to be direct and forthright when discussing with people.
I am a unique individual.
What happens to me is my own doing.

When I succeed, it is usually because of my abilities.
I enjoy being unique and different from others in many ways.

Vertical individualism(V-I)
It annoys me when other people perform better than I do.
Competition is the law of nature.
When another person does better than I do, I get tense and aroused.
Without competition, it is not possible to have a good society.
Winning is everything.
It is important that I do my job better than others.
I enjoy working in situations involving competition with others.
Some people emphasize winning; I'm not one of them.

Horizontal collectivism(H-C)
The well-being of my co-workers is important to me.
If a co-worker gets a prize, I would feel proud.
If a relative were in financial difficulty, I would help within my means.
It is important to maintain harmony within my group.
I like sharing little things with my neighbors.
I feel good when I cooperate with others.
My happiness depends very much on the happiness of those around me.
To me, pleasure is spending time with others.

Vertical collectivism(V-C)
I would sacrifice an activity that I enjoy very much if my family did not approve of it.
I would do what would please my family, even if I detested that activity.
Before taking a major trip, I consult with most members of my family and many friends.
I usually sacrifice my self-interest for the benefit of my group.
Children should be taught to place duty before pleasure.
I hate to disagree with others in my group.
We should keep our aging parents with us at home.
Children should feel honored if their parents receive a distinguished award.

E. Horizontal and Vertical Individualism and Collectivism[5]
Horizontal individualism
I'd rather depend on myself than others.
I rely on myself most of the time;
I rarely rely on others.
I often do "my own thing."
My personal identity, independent of others, is very important to me.

Vertical individualism
It is important that I do my job better than others.

Winning is everything.
Competition is the law of nature.
When another person does better than I do, I get tense and aroused.

Horizontal collectivism
If a coworker gets a prize, I would feel proud.
The well-being of my coworkers is important to me.
To me, pleasure is spending time with others.
I feel good when I cooperate with others.

Vertical collectivism
Parents and children must stay together as much as possible.
It is my duty to take care of my family, even when 1 have to sacrifice what I want.
Family members should stick together, no matter what sacrifices are required.
It is important to me that I respect the decisions made by my groups.

F. Individualism and collectivism Scale[6]
Vertical collectivism
It is important to me that I respect decisions made by my groups.
Family members should stick together, no matter what sacrifices are required.
Parents and children must stay together as much as possible.
It is my duty to take care of my family, even when I have to sacrifice what I want.

Vertical individualism
Winning is everything.
It is important to me that I do my job better than others can do it.
Competition is the law of nature.
When another person does better than I do, I get tense and aroused.

Horizontal collectivism
The well-being of my coworkers is important to me.
If a coworker gets a prize, I feel proud.
To me, pleasure is spending time with others.
I feel good when I cooperate with others.

Horizontal individualism
I often do ‘my own thing.’
I'd rather depend on myself than on others.
I rely on myself most of the time; I rarely rely on others.
My personal identity independent from others is very important to me.

G. Self-Construal Scale[7]

Interdependence Items
I have respect for the authority figures with whom I interact.
It is important for me to maintain harmony within my group.
My happiness depends on the happiness of those around me.
I would offer my seat in a bus to my professor.
I respect people who are modest about themselves.
I will sacrifice my self-interest for the benefit of the group I am in.
I often have the feeling that my relationships with others are more important than my own accomplishments.
I should take into consideration my parents' advice when making education/career plans.
It is important to me to respect decisions made by the group.
If my brother or sister fails, I feel responsible.
I will stay in a group if they need me, even when I am not happy with the group.
Even when I strongly disagree with group members, I avoid an argument.

Independence Items
I'd rather say "No" directly, than risk being misunderstood.
Speaking up during a class is not a problem for me.
Having a lively imagination is important to me.
I am comfortable with being singled out for praise or rewards.
I am the same person at home that I am at school.
Being able to take care of myself is a primary concern for me.
I feel comfortable using someone's first name soon after I meet them, even when they are much older than I am.
I prefer to be direct and forthright when dealing with people I've just met.
I act the same way no matter who I am with.
I enjoy being unique and different from others in many respects.
My personal identity, independent of others, is very important to me.
I value being in good health above everything.

H. Collectivism and Kanjin-shugi Scale [8]
Collectivism
I would rather leave my group if I have to sacrifice my self-interest for the group.
I am prepared to do things for my group at any time, even though I have to sacrifice my own interest.
I don't sacrifice self-interest for my group.
I stick with my group even through difficulties.
I think it is more important to give priority to group interests rather than to personal ones.
I respect decisions made by my group.
I don't support my group when they are wrong.

Agency
I stick to my opinions even when others in my group don't support me.

I do things in my way regardless of what my group members expect me to do.
I don't think it necessary to act as fellow group members would prefer.
I base my actions more upon my own judgments than upon the decisions of my group.
I don't change my opinions in conformity with those of the majority.
I feel uneasy when my opinions are different from those of members of my group.
I think it is desirable for the members of my group to have the same opinions.

Assertiveness
I don't say anything even when I am dissatisfied with a decision made by my group.
I often pretend to agree with the majority opinion in my group.
I state my opinions in my group only when I am confident that they are those which are endorsed by everyone .
I assert my opposition when I disagree strongly with the members of my group
I don't want to stand out in my group.

Kanjin-shugi Scale: Relatedness
I feel like doing something for people in trouble because I can almost feel their pains.
I often do what I feel like doing without paying attention to others' feelings.
I am not too concerned about other people's worries.
I am not interested in other people's business.

I. Asian Value Scale[9]
Collectivism
1.One should consider the needs of others before considering one's own needs.
2.One's achievements should be viewed as family's achievements.

Conformity to Norms
3.One need not conform to one's family's and the society's expectations. (reverse scored)
4.Following familial and social expectations are important.
5.One need not follow the role expectations (gender, family hierarchy) of one's family. (reverse scored)
6.One need not follow one's family's and the society's norms. (reverse scored)
7.One should not deviate from familial and social norms.
8.The worst thing one can do is to bring disgrace to one's family reputation.
9.Family's reputation is not the primary social concern. (reverse scored)
10.When one receives a gift, one should reciprocate with a gift of equal or greater value.

Emotional Self-Control
11.Parental love should be implicitly understood and not openly expressed.
12.One should have sufficient inner resources to resolve emotional problems.
Family Recognition Through Achievement
13.One need not achieve academically in order to make one's parents proud. (reverse scored)
14.Educational failure does not bring shame to the family. (reverse scored)

15.Occupational failure does not bring shame to the family. (reverse scored)

Filial Piety
16.Children should not place their parents in retirement homes.
17.Children need not take care of their parents when the parents become unable to take care of themselves. (reverse scored)
18.Elders may not have more wisdom than younger persons. (reverse scored)
19.One's family need not be the main source of trust and dependence. (reverse scored)

Humility
20.One should be humble and modest.
21.Modesty is an important quality for a person.
22.One should not be boastful.

J. The Asian American values scale[10]
1.Collectivism
The welfare of the group should be put before that of the individual.
One's efforts should be directed toward maintaining the well-being of the group first and the individual second.
One's personal needs should be second to the needs of the group.
The needs of the community should supersede those of the individual.
One need not always consider the needs of the group first. (Reversed order item)
The group should be less important than the individual.
One need not sacrifice oneself for the benefit of the group. (Reversed order item)

2.Conformity to Norms
One should recognize and adhere to the social expectations, norms, and practices.
One should adhere to the values, beliefs, and behaviors that one's society considers normal and acceptable.
Conforming to norms provides one with identity.
One need not blend in with society. (Reversed order item)
Conforming to norms is the safest path to travel.
Conforming to norms provides order in the community.
One should not do something that is outside of the norm.

3.Emotional Self-Control
It is better to show emotions than to suffer quietly. (Reversed order item)
One should be expressive with one's feelings. (Reversed order item)
Openly expressing one's emotions is a sign of strength. (Reversed order item)
It is better to hold one's emotions inside than to burden others by expressing them.
It is more important to behave appropriately than to act on what one is feeling.
One should not express strong emotions.
One's emotional needs are less important than fulfilling one's responsibilities.

One should not act based on emotions.

4.Family Recognition Through Achievement
One should achieve academically since it reflects on one's family.
Succeeding occupationally is an important way of making one's family proud.
Getting into a good school reflects well on one's family.
Failing academically brings shame to one's family.
One should go as far as one can academically and professionally on behalf of one's family.
One's academic and occupational reputation reflects the family's reputation.
Academic achievement should be highly valued among family members.
One's achievement and status reflect on the whole family.
Making achievements is an important way to show one's appreciation for one's family.
One's educational success is a sign of personal and familial character.
One should work hard so that one won't be a disappointment to one's family.
It is one's duty to bring praise through achievement to one's family.
Receiving awards for excellence need not reflect well on one's family. (Reversed order item)
Children's achievements need not bring honor to their parents. (Reversed order item)

5.Humility
One should be able to brag about one's achievements. (Reversed order item)
One should be able to boast about one's achievement. (Reversed order item)
One should not sing one's own praises.
One should not openly talk about one's accomplishments.
One should be able to draw attention to one's accomplishments. (Reversed order item)
Being boastful should not be a sign of one's weakness and insecurity. (Reversed order item)

K. Individualism and Collectivism Scale[11]
Individualism items
Independent
1.I don't like to rely on other people
2.What happens to me is my own doing
3.I like to act independently and take matters into my own hands
4.I try to live my life independent of others as much as possible
5.I mainly depend on myself, rarely on others
6.When facing a difficult personal problem, it is better to decide what to do yourself, than to follow the advice of others

Competitive
1.I want to be the best every time I compete
2.I feel that I have to be better than everyone else
3.I enjoy competing against others
4.I perform my best when I am competing against others
5.When another person does better than I do, I get tense and anxious

6.I feel gratified when I excel and others do not
7.I would never allow others to take the credit for something I accomplished
8.I hate to lose

Unique
1.Being distinctive is important to me
2.I intentionally do things to make myself different from those around me
3.I am a unique individual
4.I am different from others
5.I like to dress differently from others
6.The way I enjoy myself is different from others

Collectivism
1.When making decisions, it is important for me to consider the effects that my decisions have on my parents
2.When making decisions, it is important for me to take my parents' needs into account
3.When making decisions, it is important for me to take my parents' feelings into account
4.If I decided to change my job, one of the major concerns would be how this change would affect my parents
5.If I decided to get married, one of the major concerns would be how my marriage would affect my parents
6.If I moved to another city, it would be important for me to consider how my parents would be affected

Sharing positive outcome
1.I would be honored by my parents' accomplishments
2.I would feel honored if my parents received a distinguished award
3.If my parents were to have a successful career, I would be very proud of them
4.My parents would be honored if I got into a prestigious school
5.If I got a good job, my parents would be very proud of me
6.If I were successful, my parents would be honored

Sharing negative outcome
1.If my parents were caught shoplifting, I would be humiliated
2.If my parents were losers in life, I would be embarrassed
3.I would feel ashamed by my parents' misconduct
4.My misconduct would make my parents feel ashamed
5.If I lost a prestigious job, it would humiliate my parents
6.If I failed a class, it would be an embarrassment to my parents

L. the 30 Auckland Individualism and Collectivism Scale(AICS)[12]
1.I discuss job or study-related problems with my parents.
2.I consult my family before making an important decision.

3.Before taking a major trip, I consult with most members of my family and many friends.
4.It is important to consult close friends and get their ideas before making a decision.
5.Even when I strongly disagree with my group members, I avoid an argument.
6.I hate to disagree with others in my group.
7.It is important to make a good impression on one's manager.
8.In interacting with superiors, I am always polite.
9.It is important to consider the needs of those who work above me.
10.I sacrifice my self-interest for the benefit of my group.
11.I reveal personal things about myself.
12.I have the feeling that my relationships with others are more important than my own accomplishments.
13.I like to live close to my good friends.
14.To me, pleasure is spending time with my superiors.
15.To me, pleasure is spending time with others.
16.I help acquaintances, even if it is inconvenient
17.I define myself as a competitive person.
18.I enjoy working in situations involving competition with others.
19.Without competition, it is not possible to have a good society.
20.Competition is the law of nature
21.I consider myself as a unique person separate from others.
22.I enjoy being unique and different from others.
23.I see myself as “my own person”.
24.I take responsibility for my own actions
25.It is important for me to act as an independent person.
26.Being able to take care of myself is a primary concern for me.
27.I consult with my superior on work-related matters.
28.I prefer to be self-reliant rather than depend on others.
29.It is my duty to take care of my family even when I have to sacrifice what I want
30.When faced with a difficult personal problem, it is better to decide for myself, than follow the advice of others.

M. Individualism-collectivism Scale[13]
Most people see themselves as independent from others.
Most people see themselves as part of their group.

Most people enjoy being different others.
Most people enjoy being similar to from others.

Most people stress their personal accomplishments and achievements when meeting new people.
Most people stress accomplishments and achievements of their group when meeting new people.

It is important for most people to act as an independent person.
It is important for most people to act as member of their group.

When people have a need, they rely on themselves.
When people have a need, they turn to others for help.

If there is a conflict between personal values and the values of a group, most people follow their personal values.
If there is a conflict between personal values and the values of a group, most people follow the values of their group.

Most people do what is enjoyable to them personally.
Most people carry out their group obligations.

Most people pay attention to their personal contracts.
Most people pay attention to their group duties.

Most people obey their personal contracts rather than their group contracts.
Most people obey their group norms and duties rather than personal norms and duties.

Most people act in line with their rights.
Most people act in line with their group norms and duties.

Most people follow their personal attitudes.
Most people follow their group norms and rules.

Most people do their duties only if they think they will benefit from it.
Most people do their duties, even when they think they will not benefit.

When making decisions, most people are not especially sensitive to feelings of people around them.
Most people take feelings of people around them into account when making decisions.

Most people only consider needs of others in their group, if they expect something from them in return.
Most people consider needs of others in their group, even if they do not expect something from them in return.

Most people carefully calculate costs and benefits of their relationship with other people.
Most people focus on the relationship with other people without caring about associated costs and benefits.

Before helping other people, most people consider the costs of helping.
Most people generally help other people without considering costs.

Most people do not hesitate to change established relationships if the relationship is not in their best interest anymore.
Most people maintain established relationships, even if this is not in their best interest.

Most people are mainly concerned with their own personal goals.
Most people are mainly concerned with the goals of their group.

In situations of conflict between the goals of one's group and personal goals, people pursue their own goals.
In situations of conflict between the goals of one's group and personal goals, people sacrifice their own goals to achieve the goals of the group.

In cases of conflict, individuals just ignore the goals of their group and they attempt to reach their personal goals.
In cases of conflict, individuals do what the group expects and demands without opposing the will of the larger group.

The goals of individuals within a group and the goals of the group are often not compatible.
The goals of the group and the goals of individuals within groups are often compatible.

It feels natural for most people to pursue their personal goal without considering the goals of their group.
If feels natural for most people to pursue personal goals only if they do not conflict with goals of their group.

N. Personal Cultural Orientations Scale[14]
Independence (IND)
1.I would rather depend on myself than others.
2.My personal identity, independent of others, is important to me.
3.I rely on myself most of the time, rarely on others.
4.It is important that I do my job better than others.
5.I enjoy being unique and different from others in many respects.
6.I often do 'my own thing.'

Interdependence (INT)
7.The well-being of my group members is important for me.
8.I feel good when I cooperate with my group members.
9.It is my duty to take care of my family members, whatever it takes.
10.Family members should stick together, even if they do not agree.
11.I enjoy spending time with my group members.
12.Children must respect the decisions made by their parents.

Power (POW)
13.I easily conform to the wishes of someone in a higher position than mine.
14.It is difficult for me to refuse a request if someone senior asks me
15.I tend to follow orders without asking any questions.
16.I find it hard to disagree with authority figures.
17.People in higher positions have more power those in lower positions

Social Inequality (TEQ)
18.A person's social status reflects his or her place in the society.
19.It is important for everyone to know their rightful place in the society.
20.It is difficult to interact with people from different social status than mine.
21.Unequal treatment for different people is an acceptable way of life for me.
22.I believe some people have an advantage over others in every society.

Risk Aversion (RSK)
23.I tend to avoid talking to strangers.
24.I prefer a routine way of life to an unpredictable one full of change.
25.I would not describe myself as a risk-taker.
26.I do not like taking too many chances to avoid making a mistake.
27.I am very cautious about how I spend my money.
28.I am seldom the first person to try anything new.

Ambiguity Intolerance (AMB)
29.I find it difficult to function without clear directions and instructions.
30.I prefer specific instructions to broad guidelines.
31.I tend to get anxious easily when I don't know an outcome.
32.I feel stressful when I cannot predict consequences.
33.I feel safe when I am in my familiar surroundings.
34.I get confused easily when dealing with complex problems.

Masculinity (MAS)
35.Women are generally more caring than men.
36.Men are generally physically stronger than women.
37.Men are generally more ambitious than women.
38.Women are generally more modest than men.
39.Men are generally more logical than women.
40.Men are generally more aggressive than women.

Gender Equality (GEQ)
35.It is ok for men to be emotional sometimes.
36.Men do not have to be the sole bread winner in a family.
37.Men can be as caring as women.
38.Women can he as ambitious as men.
39.Men and women can be equally aggressive.

40.There is nothing that men can do but women can not.

Tradition (TRD)
47.I am proud of my culture.
48.Respect for tradition is important for me.
49.I value a strong link to my past.
50.Traditional values are important for me.
51.I care a lot about my family history.
52.I always protect my family heritage.

Prudence (PRU)
53.I believe in planning for the long term.
54.I work hard for success in the future.
55.I am willing to give up today's fun for success in the future.
56.I do not give up easily even if I do not succeed on my first attempt.
57.I plan everything carefully.
58.I consider many alternatives before making any decision.

Consumer Ethnocentrism (CET)
59.We should not buy foreign products, because it hurts our economy.
60.Only products that are unavailable in our country should be imported.
61.Purchasing foreign products allows other countries to get rich off of us.
62.It may cast me in the long run but I support my own country's products.

Consumer Innovativeness (CIN)
63.I am more interested in buying new than known products.
64.I like to buy new and different products.
65.I am usually among the first to try new products.
66.I know more than others about latest new products.

O. Modern values and Traditional values Scale[15]
I always live in accordance with my own style, regardless of what others think.
Compared with working hours, I believe leisure time is far more significant to me.
Life is just like a dream, so one should enjoy one's life now.
One should pursue the latest fashions of dress and hairstyle.
A self-employed job is more attractive than that of state-owned enterprises.
At present, going abroad is your best choice.
One should specialize, and love and focus on one's current job.
One of the principles of Lei Feng, 'it is a pleasure to help others', should be further enhanced.
One should love one's work unit as one's own home.
National needs are always superior to personal needs in choosing a job.
Some flaws exist in the system of 'Da Guo Fan', but it's safer and steadier.
Score-loading on items of secular-orientation/ideology-orientation

‘Be able to make and spend money' is a modern lifestyle.
Divorce is a normal social phenomenon.
Unemployment is a normal social phenomenon.
It is worth taking a high-risk but high-pay job.
If I had a huge amount of money, I would do nothing but enjoy my life
The range of personal income should be broadened.
Earning money is much more important than pursuing knowledge and education.
Nowadays, with money, I can do and get whatever I want.
Those who do heavy manual labour are inferior.
It is more important to find a well-reputed job than to make more money.
Happiness belongs to those who are always complacent.
Principles such as hard work and supporting a family by thrift should be further developed.

P. Collectivist Orientation Scale[16]
I view myself as a member of a social group
If I have done an excellent job, I attribute my success to collective effort
It is my duty to defend the reputation of my organization
As an employee, I have to respect decisions made by my organization
The happiness of those who are closely related to me is more important than my own happiness
What is good for my organization is also good for me
Having harmonious relationships with colleagues is my path to success
The most important thing in my life is to have good friends and easygoing colleagues
My close interpersonal relationships reflect who I am
In the workplace, disagreement should be avoided because it damages harmonious relationships with colleagues
My work success depends more on networks and relationships than on my own abilities and efforts
I cannot think of myself without relating myself to close friends and family
I never rely on my friends and colleagues to help me out with difficulties(reversed)
If the group is slowing me down, it is better to leave it and work alone(reversed)

Reference List
1.Oyserman, D.; H.M. Coon; M. Kemmelmeier. Rethinking individualism and collectivism: Evaluation of theoretical assumptions and meta-analyses. *Psychological Bulletin*, **2002**. *128*(1): p. 3-72.
2.Triandis, H.C.;Brislin, R.; Hui, C. H. Cross-cultural training across the individualism-collectivism divide. *International Journal of Intercultural Relations*, **1988**. *12(3), 269–289*.
3.Chao, R.K. Beyond parental control and authoritarian parenting style: understanding Chinese parenting through the cultural notion of training. *Child development*, **1994**. *65*(4): p. 1111-9.
4.Singelis, T.M.; Triandis, H. C.; Bhawuk, D.; Gelfand, M. J. Horizontal and vertical dimensions of individualism and collectivism: A theoretical and measurement refinement. *Cross-Cultural Research: The Journal of Comparative Social Science*, **1995**. *29(3), 240–275.*
5.Triandis, H.C.G.; M. J. Converging measurement of horizontal and vertical individualism and

collectivism. *Journal of Personality and Social Psychology*, **1998**. *74(1)*: p. 118–128.
6.Triandis, H.C. *Individualism & collectivism*. **1995:** Westview Press.
7.Singelis, T.M.; W.J. Brown. Culture, self, and collectivist communication: linking culture to individual behavior. *Human communication research*, **1995**. *21*(3): p. 354-89.
8.Kashima, Y.; S. Yamaguchi; U. Kim; S.C. Choi; M.J. Gelfand; M. Yuki. Culture, gender, and self: a perspective from individualism-collectivism research. *Journal of personality and social psychology*, **1995**. *69*(5): p. 925-37.
9.Kim, B.S.; P.H. Yang;D.R. Atkinson;M.M. Wolfe; S. Hong. Cultural value similarities and differences among Asian American ethnic groups. *Cultural diversity & ethnic minority psychology*, **2001**. *7*(4): p. 343-61.
10.Kim, B.K.; L.C. Li; G.F. Ng. The Asian American values scale--multidimensional: development, reliability, and validity. *Cultural diversity & ethnic minority psychology*, **2005**. *11*(3): p. 187-201.
11.Chen, F.F.; West, S. G. Measuring individualism and collectivism: The importance of considering differential components, reference groups, and measurement invariance. *Journal of Research in Personality*, **2008**. *42(2), 259–294*.
12.Shulruf, B.; J. Hattie;R. Dixon. Development of a new measurement tool for individualism and collectivism. *Journal of Psychoeducational Assessment*, **2007**. *25*(4): p. 385-401.
13.Tardif-Williams, C.Y.; L. Fisher. Clarifying the link between acculturation experiences and parent-child relationships among families in cultural transition: The promise of contemporary critiques of acculturation psychology. *International Journal of Intercultural Relations*, **2009**. *33*(2): p. 150-161.
14.Sharma, P. Measuring personal cultural orientations: scale development and validation. *Journal of the Academy of Marketing Science*, **2010**. *38*(6): p. 787-806.
15.Sun, J.; X. Wang. Value differences between generations in China: a study in Shanghai. *Journal of Youth Studies*, **2010**. *13*(1): p. 65-81.
16.Van de Vliert, E.; H. Yang; Y. Wang; X.-p. Ren. Climato-Economic Imprints on Chinese Collectivism. *Journal of Cross-Cultural Psychology*, **2013**. *44*(4): p. 589-605.
